# Enhanced Osteogenesis and Antibacterial Properties of Ketoprofen-Loaded MgCu-MOF74-Coated Titanium Alloy for Bone Implant

**DOI:** 10.3390/jfb16060222

**Published:** 2025-06-14

**Authors:** Ziqing Duan, Yifeng Yao, Jiamin Liu, Yanni Tan, Qingge Wang, Man Fang, Aqsa Kanwal, Shuqiao Cheng, Juan Huang, Hong Wu

**Affiliations:** 1 State Key Laboratory of Powder Metallurgy, Central South University, Changsha 410083, China; 223311010@csu.edu.cn (Z.D.); 243311022@csu.edu.cn (Y.Y.); liujiamin_csu@163.com (J.L.); tanyanni@csu.edu.cn (Y.T.); 213301043@csu.edu.cn (Q.W.); fangman@csu.edu.cn (M.F.); aqsakanwal@csu.edu.cn (A.K.); 2 Department of Pharmacy, Xiangya Hospital, Central South University, Changsha 410008, China; 3 Department of Breast Surgery, Xiangya Hospital, Central South University, Changsha 410008, China; 404369@csu.edu.cn

**Keywords:** titanium alloy, metal–organic framework, surface modification, antibacterial property, osseointegration

## Abstract

To address the dual clinical challenges of poor osseointegration and inadequate analgesia caused by postoperative infections in traditional titanium implants, this study proposes a multifunctional synergistic strategy based on metal—organic frameworks (MOFs). By integrating drug-controlled release and ionic microenvironment regulation, it constructs a titanium-based implant coating system with antibacterial and bone-regenerative properties. Ketoprofen, a drug with excellent analgesic properties, was loaded into MgCu-MOF74 powder, and the Ket@MgCu-MOF74 powder was successfully anchored onto the surface of the titanium alloy through dopamine-mediated adhesion. The maximum load of ketoprofen to MgCu-MOF74 is 18.55%, and it has a good controllable release effect. The results showed that MgCu-MOF74/Ti and Ket@MgCu-MOF74/Ti coatings enhanced osteogenic performance by promoting alkaline phosphatase activity, collagen secretion, and extracellular matrix mineralization. Additionally, the release of Mg^2+^ and Cu^2+^ created an alkaline environment, providing antibacterial properties. In summary, the MOF enabled the controlled release of ketoprofen, and the composite coating can improve osteogenic differentiation of osteoblasts and enhance the antibacterial properties of titanium alloy implants.

## 1. Introduction

Titanium alloys are extensively used in orthopedic implants due to their exceptional mechanical properties [1,2] and favorable biocompatibility [3,4,5]. Despite these benefits, conventional titanium alloy implants face significant challenges in clinical applications and poor osseointegration ability. Therefore, it is essential to develop implants that not only exhibit strong early-stage antibacterial properties but also significantly promote bone regeneration [6].

To enhance the antibacterial ability and osteogenic properties of implant devices, researchers have extensively explored surface modifications of titanium and its alloys [7,8,9,10,11,12]. Although antibiotics and antibacterial peptides have been successfully applied to titanium surfaces, long-term use can lead to the development of antibiotic resistance in bacteria, thereby reducing the effectiveness of antibacterial action [13,14]. The incorporation of metal ions (e.g., Mg^2+^, Zn^2+^, and Cu^2+^) can effectively overcome resistance and enhance the growth and differentiation of osteoblasts while also reducing inflammation [15].

Mg^2+^ ions serve as vital trace elements within the human body, fulfilling crucial functions in sustaining numerous essential biological processes and facilitating tissue repair [16]. Mg^2+^ ions are the most prevalent divalent cations found within cells, playing a crucial role in various cellular processes such as signaling, transduction, growth, metabolism, and proliferation [17]. Research indicates that Mg^2+^ could enhance the osteogenic differentiation of MSCs by increasing autophagic activity [18]. Another work found that optimal concentrations of Mg^2^⁺ facilitated the activation of Ca ion channels in cell membranes, promoting calcium deposition necessary for bone development [19]. Furthermore, the angiogenic and anti-inflammatory characteristics of Mg^2+^ provide extra support in multiple bone regeneration scenarios [20,21]. As a result, Mg^2+^ serves as a significant factor in bone formation, highlighting the extensive application of Mg-based materials in tissue engineering for bone repair.

Metal–organic frameworks (MOFs) are distinguished as 3D nanomaterials, which are crystalline or amorphous coordination compounds formed through the self-assembly of organic ligands with metal ions or clusters [22]. Due to their distinctive architecture, MOFs exhibit remarkable properties suitable for biomedical applications, including a high specific surface area, substantial porosity, chemical stability, and biocompatibility [23]. Numerous studies have indicated the extensive use of biocompatible Mg-MOF materials across diverse biomedical fields, including drug delivery, disease treatment, and tissue repair [24,25]. Moreover, through the regulated degradation and sustained release of bioactive ions, MOFs demonstrate therapeutic functionalities while promoting tissue repair processes.

Although Mg-MOF74 offers several benefits, its poor stability in water-based environments restricts its practical applications. The incorporation of Cu^2+^ significantly influences the bioactivity and antibacterial properties of Mg-MOF74 coatings [26]. Notably, compared to conventional Mg-MOF74, Cu^2+^ doping markedly enhances water stability, which is crucial for physiological durability [27]. Introducing Cu^2+^, a metal ion known for its varied biological roles, can enhance the coating’s antibacterial effectiveness [28,29]. Cu^2+^ disrupts bacterial cell membranes, hinders intracellular enzyme functions, and promotes the production of reactive oxygen species, thereby inhibiting the growth of harmful microorganisms, reducing infection risk, and aiding in wound healing. Additionally, Cu^2+^ facilitates angiogenesis, an essential process in bone tissue engineering that speeds up bone tissue repair and regeneration [30]. Moreover, the addition of Cu^2+^ can also impact the degradation behavior and mechanical characteristics of Mg-MOF74 coatings [31]. Research indicates that Cu^2+^ may modify the degradation rate of the coating, influencing the release of Mg^2+^ and other critical ions [26]. Furthermore, the electrochemical properties of Cu^2+^ might alter the electrochemical stability and corrosion resistance of the coatings, factors critical to their long-term durability in physiological conditions [32,33]. In a previous study, Liu et al. [34] synthesized MgCu-MOF74 particles with varying Cu^2+^ concentrations using a one-step hydrothermal method. Systematic evaluation examined their structural characteristics, aqueous stability, osteogenic potential, and antibacterial capabilities. The results demonstrated that Cu^2+^ doping significantly enhanced the water stability, cellular viability, and antibacterial performance of MgCu-MOF74, highlighting its excellent osteogenic and antibacterial properties. Therefore, we propose developing an MOF coating on titanium surfaces to release a substantial amount of metal ions for early postoperative bacterial elimination without compromising subsequent osteogenesis.

In titanium-based implants, effective osseointegration and infection prevention were achieved by modifying the titanium surface with bioactive materials [35]. Polydopamine (PDA) was used as an adhesive layer due to its strong adhesion, biocompatibility, and ability to form stable coatings [36,37]. Inspired by mussel adhesive proteins, PDA polymerizes under mild conditions, promoting uniform MOF deposition while preserving biological functionality. This approach enhances the mechanical stability and bioactivity of the coating, ensuring the effective delivery of therapeutic agents and improving cell adhesion and proliferation for better implant performance [38]. Ketoprofen (Ket) is a propionic acid NSAID that non-selectively inhibits cyclooxygenase to attenuate inflammation and relieve pain following bone injury and joint inflammation [39]. However, the prolonged use of such NSAIDs may lead to delayed healing and reduced mineralization of new bone [40]. Compared to oral administration, in situ application through implantation of drug carriers for controlled drug release enables better recovery [41,42]. Ketoprofen was selected for its intermediate molecular weight, pKa, and balanced lipophilicity, which enable strong hydrogen-bonding and π–π stacking interactions with MgCu-MOF74 pore walls. These interactions yield a high loading efficiency and a controlled biphasic release profile, thereby avoiding the toxic burst release observed with more hydrophobic NSAIDs [43]. Therefore, drug-loaded biocoatings are emerging as a superior alternative to traditional implants and clinical therapies [44].

Current titanium implant surface modifications face several critical limitations, and conventional metal ion coatings exhibit inadequate early-stage antibacterial efficacy, while antibiotic-based approaches risk bacterial resistance development. Additionally, there is an absence of comprehensive strategies that simultaneously achieve robust early-stage infection control and sustained osteogenic promotion, along with an insufficient understanding of coating stability and controlled release mechanisms in physiological environments.

To address these challenges, we successfully synthesized MgCu-MOF74 via a hydrothermal method and applied Ket@MgCu-MOF74 coatings to titanium alloy surfaces using dopamine-mediated adhesion. This study systematically investigated the loading and release mechanisms of ketoprofen within the bimetallic MgCu-MOF74 framework, the degradation behavior, the ionic release processes of MgCu-MOF74/Ti and Ket@MgCu-MOF74/Ti coatings under simulated physiological conditions, and their antibacterial and osteogenic performance.

## 2. Materials and Methods

### 2.1. Reagents and Chemicals

Magnesium nitrate hexahydrate (Mg(NO_3_)_2_·6H_2_O, 99%) was obtained from Shanghai Experimental Reagent Co., Ltd. (Shanghai, China). Copper nitrate hexahydrate (Cu(NO_3_)_2_·3H_2_O, 99%), 2, 5-dihydroxyterephthalic acid (DHTA, 98%) and N, N-dimethylformamide (DMF, 99%) were supplied by Beijing Labgic Technology Co., Ltd. (Beijing, China). Ketoprofen (C_16_H_14_O_3_), hydrochloric acid (HCl, 99.7%), acetone (C_3_H_6_O, 99.7%), ethanol (C_2_H_5_OH, 99.9%), sodium hydroxide (NaOH, 99.7%), dopamine (C_8_H_11_NO_2_, 98%), and Tris-HCl powder were purchased from Hubei Xinkang Pharmaceutical Chemical Co., Ltd. (Tianmen, China).

### 2.2. Fabrication of Ket@MgCu-MOF74

**Figure 1:**
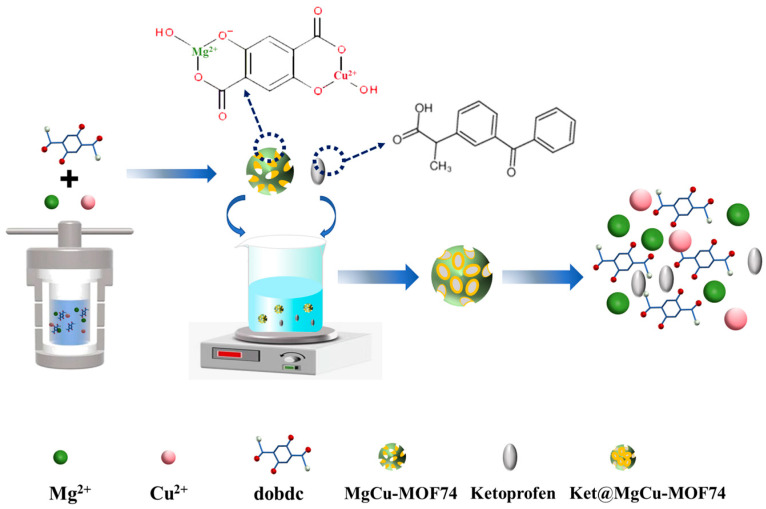
Schematic diagram of Ket@MgCu-MOF74.

A schematic outlining the preparation of Ket@MgCu-MOF74 is shown in Figure 1. First, MgCu-MOF74 was synthesized by the hydrothermal method by adding 158.4 mg of DHTA to a mixture of 64.5 mL of DMF, 4.3 mL of ethanol, and 4.3 mL of ultrapure water. The mixture of 2.5 mmol Mg(NO_3_)_2_·6H_2_O and 0.1 mmol Cu(NO_3_)_2_·3H_2_O was dissolved in the above solution and combined with magnetic agitation and ultrasound until completely dissolved. The sample underwent thermal processing in a muffle furnace at 125 °C for 24 h under sealed high-pressure conditions, followed by a six-hour spontaneous cooling phase. Post-synthesis processing involved retrieval of the resultant products, which were purified through triplicate washing cycles using dimethylformamide (DMF) and ethanol, before being subjected to vacuum dehydration at 60 °C lasting 72 h. The crude MgCu-MOF74 was soaked in formaldehyde for three days, and the formaldehyde was replaced every 12 h. The impregnated powder was placed in a drying oven at 200 °C and dried under vacuum for 12 h. In total, 1.5 g of ketoprofen was soaked in 10 mL of ethanol and stirred at 600 r/min at room temperature. Following the complete dissolution of ketoprofen, 50 mg of MgCu-MOF74 was introduced into the solution and subjected to continuous agitation (600 r/min) under ambient conditions for 24 h. Post-centrifugal separation, the supernatant was discarded to retain the precipitated composite particles at the bottom. The synthesized composite particles underwent purification through sequential methanol and deionized water rinses for three cycles, followed by oven drying at 120 °C for 24 h. The name of the new product was Ket@MgCu-MOF74.

### 2.3. Fabrication of Drug-Loaded Coating on Titanium Alloy Surface

The MgCu-MOF74 coating was fabricated on the titanium alloy surface through the impregnation method, relying on the adhesion and deposition behavior of the catechol and phenethylamine groups in PDA. First, the commercial titanium alloy piece sized 13.5 mm × 13.5 mm × 1.5 mm was polished successively with diamond sandpapers of 400, 600, 800, 1000, 1200, 1500, and 2000 mesh. Then, it was ultrasonically cleaned for 15 min each in ethanol, acetone, and deionized water in sequence. Finally, it was put into a vacuum drying oven for later use. The dopamine was dissolved in a 10 mM Tris buffer solution, and the pH was adjusted to 8.5, resulting in a final dopamine concentration of 2 mg/mL. Three groups of titanium matrix were placed in PDA solution, MgCu-MOF74 working liquid, and Ket@MgCu-MOF74 working liquid, respectively. Next, the above samples were placed under ultraviolet light for 12 h. Finally, the samples cleaned by anhydrous ethanol were dried. The obtained samples were named Ti, PDA, MgCu-MOF74/Ti, and Ket@MgCu-MOF74/Ti.

### 2.4. Microstructural Characterization

Scanning electron microscopy (SEM, FEI Quanta FEG250, Hillsboro, OR, USA) and energy dispersive spectrometry (EDS, EDAX, Mahwah, NJ, USA) were used to evaluate the microscopic morphology and element distribution of the samples. Before observation, all samples should be sprayed with gold. X-ray diffractometer (XRD, MAX-2250, Tokyo, Japan) was used to detect the crystal structure of different samples. Elemental chemical states were characterized by utilizing X-ray photoelectron spectrometer (XPS, ESCALAB250Xi, Waltham, MA, USA). The composition of functional groups on the surface of the sample was investigated by Fourier transform infrared spectroscopy (ATR-FTIR, Nicolet iS50, Madison, WI, USA). The thermal stability of the samples was evaluated by thermogravimetry (TG, TGA 550, New Castle, DE, USA). Test atmosphere was nitrogen, and the measured temperature was 0–700 °C.

### 2.5. Ketoprofen Loading and Release Behavior

Ketoprofen loading and release were determined by HPLC (LC1260 Infinit II, Santa Clara, CA, USA). Firstly, 5 mg of Ket@MgCu-MOF74 was stirred into 5 mL of 0.1 M NaOH solution at room temperature for 30 min and then centrifuged. A 1 mL aliquot of the supernatant was mixed with 1 mL of methanol, filtered through a 0.45 μm membrane filter, and analyzed by HPLC. Drug loading efficiency (*DLE*) was calculated according to the ketoprofen standard curve.

*DLE*% = *Mt*/*Ms* × 100%

where *Mt* is the total mass of ketoprofen, and *Ms* is the total mass of MgCu-MOF74.

Ket@MgCu-MOF74 (100 mg) was dispersed in 50 mL PBS and subjected to continuous agitation (300 r/min) under ambient conditions over 48 h. At regular intervals, 2 mL of supernatant was extracted, with simultaneous replenishment of equivalent volumes of PBS to maintain the balance of the solution. The ketoprofen release from Ket@MgCu-MOF74 in PBS solution was determined by high-performance liquid chromatography. Finally, the ketoprofen release percentage (*CR*%) was calculated by the following equation:

*CR*% = *Mr*/*Mt* × 100%

where *Mr* is the released mass of ketoprofen, and *Mt* is the total mass of loaded ketoprofen.

### 2.6. Stability of the Coatings

In order to obtain the release trend of Mg^2+^ and Cu^2+^ in MgCu-MOF74/Ti and Ket@MgCu-MOF74/Ti sample groups at different time nodes and under different physiological environments, the samples were positioned in 24-well plates. After adding PBS (initial pH values of 6.5 and 7.4, respectively), the immersed samples were placed in a constant temperature environment at 37 °C. At a specific time node, all the liquid in the plate was transferred and divided, and a new solution was added to each well of the plate for immersion. The release of Mg^2+^ and Cu^2+^ was determined by an inductively coupled plasma emission spectrometer (ICP-AES, SPECTRO BLUE SOP, Kleve, Germany). In order to explore the change in the pH value of the samples in different culture environments, the four groups of coating samples were placed in DMEM medium and Luria–Bertani (LB) liquid medium, respectively, and the pH value of the supernatant was measured over a specific period of time. Electrochemical experiments of all samples were tested using an electrochemical workstation (CS310H, Potentionstat/Galvanostat, Wuhan, China) in the 0.9% NaCl solution.

### 2.7. Material Surface Wet Ability Test

Wettability characterization of the composite coatings was conducted using a contact angle tester (CAM; OCA 25LHT, Data Physics Instrument GmbH, Stuttgart, Germany). The test sample was placed on a horizontal platform, the distance between the sample platform and the water outlet was adjusted, and the deionized water was slowly dropped onto the sample. After the droplet stabilized, images were captured to determine each sample’s contact angle measurements. Three distinct surface loci per sample were analyzed through randomized coordinate selection, with arithmetic mean values calculated for statistical reliability.

### 2.8. In Vitro Experiments

#### 2.8.1. Cell Culture

SaOS-2 human osteosarcoma cells were purchased from Shanghai Hengya Biotechnology Co., Ltd. (Shanghai, China) After thawing, SaOS-2 cells were distributed in 84% McCoy’s 5A (Gibco, Grand Island, NY, USA) medium, and 15% fetal bovine serum (FBS, Gibco, Grand Island, NY, USA) and 1% penicillin/streptomycin double antibody (PS, Gibco, Grand Island, NY, USA) were mixed in a complete medium. For the osteogenic medium, 50 μg/mL of ascorbic acid, 10 mM of sodium β-glycerophosphate, and 10^−7^ M dexamethasone were incorporated into the aforementioned culture medium.

An extract solution was used to evaluate the in vitro cell experiments of coating samples, comprehensively assessing the impact of chemicals released during sample use on cells. The specimens were positioned on a sterile workbench and underwent UV light sterilization for a duration of three days. Then, the metal block samples were evenly coated with polydimethylsiloxane (PDMS, a kind of biocompatible silica gel) except the test surface and cured at 60 °C for 3 h. The samples were soaked in a culture medium with a specific surface area to medium ratio of 1.25 cm^2^/mL in a humid atmosphere of 37 °C and 5% CO_2_. After 24 h, the extracted solution was centrifuged to remove impurities and stored at 4 °C.

#### 2.8.2. Cell Proliferation

The Cell Counting Kit-8 (CCK-8) assay was used to quantify the viability of SaOS-2 cells. The cells were co-cultured with the sample extracts for 1, 3, and 5 days. Then, each well was filled with complete medium containing 10% CCK-8 solution and incubated at 37 °C for 30 min. A microplate reader (Perkin-Elmer, Waltham, MA, USA) was employed to measure the optical density at 450 nm. Moreover, live cell staining was used to evaluate the viability of osteoblasts. After being co-cultured with different sample extracts for 1, 3, and 5 days, the cells were stained with calcein-AM in the dark at 37 °C for 15 min.

#### 2.8.3. ALP Activity

For alkaline phosphatase (ALP) activity, SaOS-2 cells were implanted into 24-well plates and then lysed in 200 μL of RIPA lysis buffer for 15 min at 4 °C. The content of protein was measured using a BCA assay kit (Bio-Rad, Hercules, CA, USA). Quantitative ALP activity was assessed with an ALP activity assay kit (Beyotime, Shanghai, China) per the manufacturer’s instructions and then standardized relative to protein content. For qualitative ALP activity analysis, 150 μL of paraformaldehyde (PFA) was added to each well after washing with PBS to fix cell morphology, and staining was performed using ALP staining kit (Beyotime, Shanghai, China). Observation and imaging were performed using fluorescence microscopy (DFC420C, Wetzlar, Germany).

#### 2.8.4. COL Secretion

After co-culture with sample extracts for a specific time, the SaOS-2 cells were rinsed with PBS, and the cell morphology was fixed with PFA. Subsequently, the cells were incubated with Sirius Red (Sigma, St. Louis, MI, USA) at room temperature for 10 h away from light. The cells were rinsed with PBS to terminate the staining. Observation and imaging were performed using a fluorescence microscope. The stain was removed by using a 1:1 mixture of 0.2 M NaOH and methanol for 30 min. After that, a microplate reader was used to measure the absorbance of the solution at 520 nm.

#### 2.8.5. Mineralization

After co-culturing SaOS-2 cells with sample extracts for 14 days, the cells were rinsed with PBS and fixed with 150 μL of paraformaldehyde to preserve their morphology. Then, 200 μL of alizarin red dye solution (Sirius Red, Sigma, St. Louis, MI, USA) was added and incubated at room temperature for 1 h away from light, followed by PBS rinsing to terminate the staining. Observation and imaging were performed using the fluorescence microscope. The stain was eluted with a specific concentration of sodium dihydrogen phosphate solution for 20 min, and the absorbance of the eluent at 570 nm wavelength was measured with the microplate reader.

### 2.9. Antibacterial Activity

*E. coli* (ATCC25922) was used to assess the antibacterial activity of the specimens. Antibacterial efficacy was qualitatively determined by measuring inhibition zones against *E. coli*. Experimental protocols comprised co-incubating test specimens with 100 μL of bacterial inoculum (10^7^ CFU/mL) for 12 h under standard culture conditions. Antibacterial efficacy was evaluated through plate inoculation assays. And quantitative analysis employed 24-well plates containing 1 mL bacterial broth (10^7^ CFU/mL) incubated with the samples for 12 h, followed by serial dilution to 4000 CFU/mL. Subsequently, 100 μL aliquots from each experimental group were plated on LB solid medium. Antibacterial performance was calculated using the following computational formula:

Antibacterial rate = (1 − *N_x_*/*N_b_*) × 100%

where *N_x_* is the number of colonies on the solid medium of Ti, and *N_b_* is the number of colonies on the solid medium of PDA, MgCu-MOF74/Ti, and Ket@MgCu-MOF74/Ti, respectively.

SEM was used to observe the bacterial adhesion morphology. Samples inoculated with *E. coli* cultured (10^7^ CFU/mL) underwent 12 h incubation at 37 °C, followed by gentle PBS washing and chemical fixation in 2.5% glutaraldehyde for 30 min under ambient conditions. For dehydration, the samples were successively immersed in ethanol solutions of escalating concentrations (30%, 50%, 70%, 90%, 95%, and 100%), staying in each solution for 10 min.

### 2.10. Statistical Analysis

Each experiment was conducted with a minimum of three repetitions, with quantitative data expressed as mean ± SD. Statistical analyses employed single-factor ANOVA with the LSD test, where * p < 0.05 and ** *p* < 0.01 were considered statistically significant and highly significant, respectively.

## 3. Results and Discussion

### 3.1. Characterization of Ket@MgCu-MOF74

**Figure 2:**
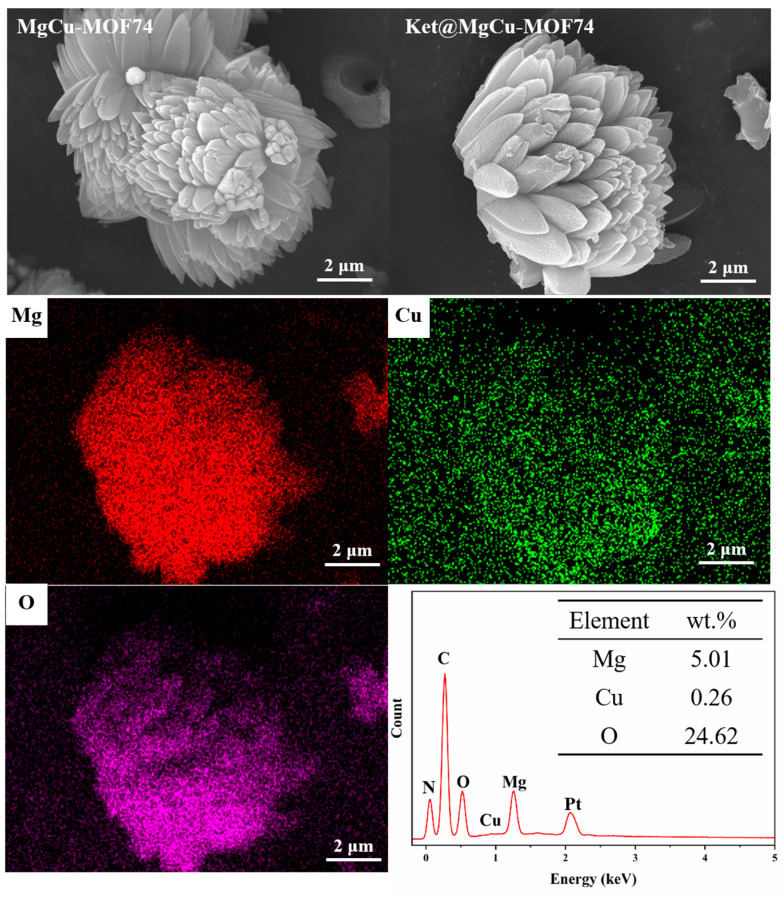
Surface morphology and EDS spectra of Ket@MgCu-MOF74.

Figure 2 shows the morphology of Ket@MgCu-MOF74. The distinctive coordination geometry of MOF-74 crystalline frameworks engenders hexagonal lattice arrangements [45], facilitating spontaneous morphogenesis into diverse architectures including cauliflower, needle, pike, bullet shell, etc. [46]. It can be found that the morphology of Ket@MgCu-MOF74 is the same as that of MgCu-MOF74, which is in the shape of a multi-petal cauliflower, only that its surface becomes relatively rough, suggesting that ketoprofen loading does not destroy its basic structure. In addition, the EDS results further indicate that Mg^2+^ and Cu^2+^ are uniformly distributed in the Ket@MgCu-MOF74 structure.

**Figure 3:**
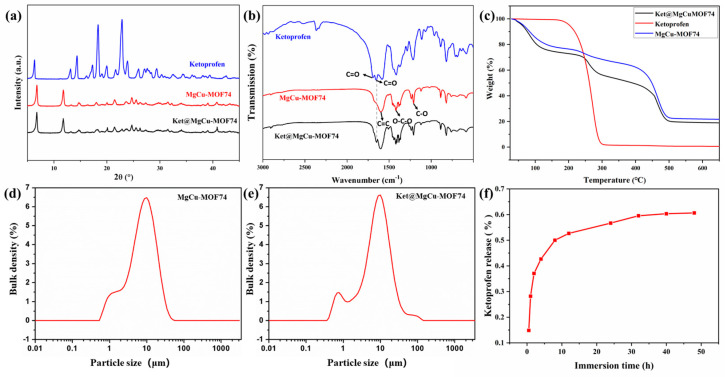
(**a**) XRD patterns, (**b**) FTIR spectra, (**c**) TG patterns, particle size distribution of (**d**) MgCu-MOF74 and (**e**) Ket@MgCu-MOF74 samples, (**f**) release curve of ketoprofen.

The XRD spectra of the synthesized samples are shown in Figure 3a. The XRD spectra of MgCu-MOF74 show significant diffraction peaks, which indicate the good crystallinity of MgCu-MOF74. Significant diffraction peaks at 2θ = 6.8° and 11.8°, which correspond to (210) and (300) planes of MOF-74 crystals, were observed in previous studies [47,48]. Compared with MgCu-MOF74, the two diffraction peaks of Ket@MgCu-MOF74 at the (210) and (300) crystal faces are slightly shifted to the right, and the peak intensity ratio, I210/I300, is increased from 1.30 to 1.41. The spectroscopic evidence confirms the effective entrapment of ketoprofen within the crystalline MgCu-MOF74. In the XRD spectra of Ket@MgCu-MOF74, ketoprofen did not show any obvious characteristic diffraction peaks, which may be due to the fact that ketoprofen was encapsulated in the MgCu-MOF74 carrier or its content was low [49].

The FTIR spectra of powders (Figure 3b) show the molecular structure and chemical bonding. According to the coordination properties of MOF materials, most of the activation modes in the vibrational spectra of MOFs above the 700 cm^−1^ region belong to organic ligands in the structure [50]. The peak at 1595 cm^−1^ is ascribed to the C=C bond vibration in the benzene ring. The 1420 cm^−1^ peak is associated with the -O-C-O group in the carbonyl structure, while the 1211 cm^−1^ peak indicates the vibration of the C-O bond [51]. Within this structure, each Mg^2+^ and Cu^2+^ coordinates with five oxygen atoms: three with carboxyl groups and two with hydroxyl groups, thereby establishing a solitary open metallic locus. This unoccupied metal locus manifests efficaciousness in hosting adsorption processes for assorted guest molecular entities like water, gaseous compounds, and DMF. During the thermal process, solvent molecules like DMF and water were easily adsorbed into MgCu-MOF74 via the open site. However, the DMF peak at approximately 1654 cm^−1^ was absent in its infrared spectra, indicating that methanol washing and heating to 200 °C effectively removed these solvents [52]. A characteristic peak originating from the stretching vibration of C=O in the carboxyl group was found at 1693 cm^−1^ of the ketoprofen spectrum, and a characteristic C=O peak originating from the keto group was found at 1651 cm^−1^ [53]. After loading ketoprofen, the characteristic peak originating from the C=O stretching in the keto group was found at 1651 cm^−1^ of the Ket@MgCu-MOF74 spectrum, whereas the C=C vibration peak belonging to the benzene ring at 1595 cm^−1^ moved to 1603 cm^−1^. These results suggest the successful loading of ketoprofen.

Figure 3c shows the TG results of MgCu-MOF74 and Ket@MgCu-MOF74. The results show that the collapse temperatures of MgCu-MOF74 and Ket@MgCu-MOF74 are both around 450 °C, indicating that the drug load does not reduce the stability of Ket@MgCu-MOF74, and the thermal stability of the frames is good. At the same time, it was found that the weight loss of both of them decreased in three stages. The quality decline in the second stage is different, mainly due to the breakdown of drugs in Ket@MgCu-MOF74. The difference in the weight loss rates between MgCu-MOF74 and Ket@MgCu-MOF74 during the second stage of thermal decomposition is primarily attributed to the thermal degradation of the drug ketoprofen loaded within the framework. The rapid decomposition of ketoprofen molecules produced volatile byproducts, accelerating the weight loss in Ket@MgCu-MOF74. Additionally, the interaction between the drug and the MOF may alter the decomposition pathway and reduce the activation energy required for thermal breakdown, further contributing to the faster weight loss rate compared to the pristine MgCu-MOF74.

Figure 3d,e demonstrate the particle size distribution of MgCu-MOF74 and Ket@MgCu-MOF74. This indicates that the loading of ketoprofen has little effect on the particle size of MOF, with approximately 32% of the particles falling within the range of 3 to 4 μm.

In the context of drug delivery systems, a key parameter is the maximum drug loading capacity. The maximum quantity of ketoprofen loaded by MgCu-MOF74 was 18.55%. The release behavior of ketoprofen in Ket@MgCu-MOF74 in PBS was investigated (Figure 3f). The release of ketoprofen occurred in two phases: the first phase was a rapid release within the initial 12 h, reaching approximately 85% release by 12 h. Clinically, this burst coincides with the period of peak postoperative pain and thus provides immediate local analgesia [54]. And the second phase was a slow-release process within 12–48 h, which showed a good controlled release.

A previous study has indicated that the drug release process depends on three key factors [55]: surface physical adsorption onto MOFs, interactions between drug molecules and pore walls (e.g., hydrogen bonding), and the pore dimensions of the carrier. Drugs adsorbed on the surface and within pores are released rapidly, whereas those trapped in pore structures through adsorption and hydrogen bonding exhibit slow release. In this study, the first 12 h of rapid release likely resulted from ketoprofen desorbing from the hexagonal honeycomb-like pores. The subsequent 12–48 h slow release primarily stemmed from the disruption of hydrogen bonds between ketoprofen and pore walls, along with degradation of the metal–organic framework structure.

### 3.2. Physical and Chemical Properties of the Coating

#### 3.2.1. Morphology, Phase, and Chemical Composition

**Figure 4:**
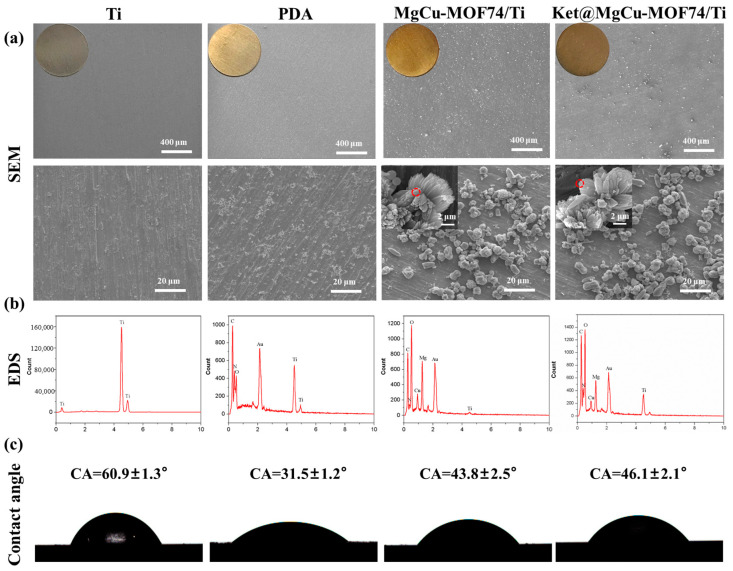
(**a**) SEM, (**b**) EDS, and (**c**) contact angle of Ti, PDA/Ti, MgCu-MOF74/Ti, and Ket@MgCu-MOF74/Ti.

Figure 4a displays the surface morphologies of the Ti, PDA/Ti, MgCu-MOF74/Ti, and Ket@MgCu-MOF74/Ti samples. The surface of the different titanium flakes exhibits distinct color changes. The sanded titanium exhibited a mildly rough surface with faint scratches. After PDA treatment, a large number of white flocculant crystals appeared on the surface of the titanium sheet, while the surface of the titanium sheet doped with MgCu-MOF74 and Ket@MgCu-MOF74 powder showed obvious fusiform crystal particles. It is noteworthy that the Ket@MgCu-MOF74 sample still exhibits a rougher surface than the MgCu-MOF74 control at higher magnification, which aligns with the previous powder SEM findings.

Figure 4b displays the EDS spectra of the samples. The results indicated that the peaks of Mg and Cu elements appeared on the surface of titanium flakes after loading powder particles. It can be concluded that, after impregnating the titanium matrix in the solution mixed with MgCu-MOF74 powder and PDA, the PDA reacted with the surface of MOF74 powder particles in the process of oxidation self-polymerization and adhesion to the titanium matrix and then adhered to the surface of the matrix. There was no significant change in the powder morphology, elemental type, or chemical composition, which is in agreement with the previous powder results.

As shown in Figure 4c, the contact angles of Ti, PDA, MgCu-MOF74/Ti, and Ket@MgCu-MOF74/Ti were about 58.4°, 31°, 42.8°, and 45.2°, respectively. The results showed that the surface wettability of titanium tablets changed significantly after different coating treatments. Compared with the Ti surface, the contact angle of materials treated with PDA and MOF powder was significantly reduced. This is because PDA contains a large number of phenolic hydroxyl (-OH) and amino (-NH_2_) groups, which introduced the hydrophilic groups to the surface of titanium alloy in the process of self-polymerization to form PDA, thus improving the hydrophilic properties of the surface of titanium sheets. The surface roughness of MOF material increases due to the presence of powder particles, and MOFs additionally exhibit excellent hydrophilic properties owing to their high specific surface area and numerous hydrophilic groups, including hydroxyl (-OH) and ester (-COOR) moieties within the framework [56]. The presence of ketoprofen has little effect on the hydrophilicity of the coating. In general, the construction of micro-nanostructures on the surface of medical metal implants is an effective means to improve their hydrophilicity.

**Figure 5:**
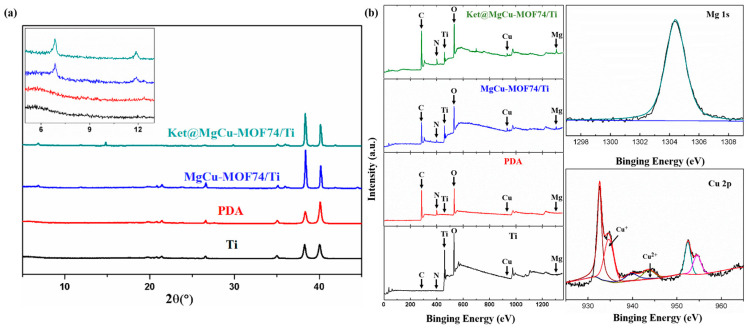
(**a**) XRD patterns of different samples, (**b**) XPS survey spectra of different samples, and high-resolution spectra of Mg1s and Cu2p in Ket@MgCu-MOF74/Ti.

Figure 5a shows the crystal structures of the different coated samples, and the results indicate that the presence of dopamine does not affect the crystal structures of the materials, and the XRD spectra of the MgCu-MOF74/Ti and Ket@MgCu-MOF74/Ti coated samples showed a distinctive characteristic peak at the position of 6.8°, which is further evidence that the dopamine has successfully bonded the MOF74 particles to the Ti surface.

Figure 5b shows the full XPS spectra of the four samples. There are no C, N, Mg, or Cu peaks on the surface of the Ti sample, but there are obvious Ti and O peaks. Normally, the surface of titanium does not contain C and N elements, but the titanium alloy is easily oxidized in the air, and, thus, the peaks of O elements appear in the XPS spectra. The main constituent elements of dopamine are C, N, and O. Therefore, the deposition of dopamine on the surface of the Ti sample introduced additional C and N elements. On the surface of the PDA coatings with immobilized MgCu-MOF74 and Ket@MgCu-MOF74, peaks of Mg and Cu elements appeared, which is in agreement with the results of Figure 4b, indicating that the PDA successfully adhered the MgCu-MOF74 and Ket@MgCu-MOF74 powders to the Ti surface. Gaussian fitting of the XPS high-resolution spectra of Mg1s and Cu2p of the Ket@MgCu-MOF74/Ti samples revealed that a small amount of Cu^2+^ was reduced to Cu^+^, which may be caused by a reduction in some Cu^2+^ in the MgCu-MOF74 particles due to the reducing nature of the phenolic hydroxyl group in PDA.

#### 3.2.2. Stability of the Coatings

**Figure 6:**
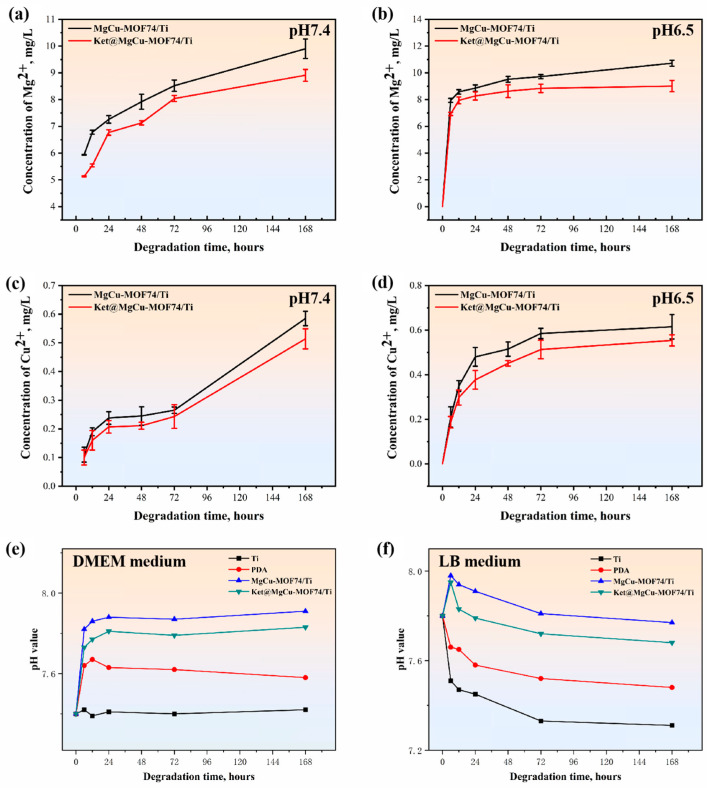
Cumulative release profiles of Mg^2+^ (**a**,**b**) and Cu^2+^ (**c**,**d**) from samples in different physiological environments (*n* = 3), and pH value of samples in (**e**) cell culture medium and (**f**) bacterial medium.

In order to analyze the sensitivity of the prepared coatings to acidic and alkaline environments, solutions with pH 7.4 and 6.5 were selected for this study. The degradation behaviors of MgCu-MOF74/Ti and Ket@MgCu-MOF74/Ti are shown in Figure 6a–d. The metal ion release trends of the two groups of samples did not differ much. In the physiological environment of pH 7.4, both samples showed a continuous ion release pattern from day 1 to day 7. Faster degradation occurred in both sample groups in the pH 6.5 microenvironment compared to pH 7.4. During the first 24 h, both sample groups showed a rapid release of metal ions. Subsequently, the rate of ion release gradually slowed down. The total Cu^2+^ released over seven days remained below 25 µM, substantially under the 100 µM cytotoxic threshold for osteoblasts, indicating that copper ion release from our coatings is maintained within a safe therapeutic range [57].

Moreover, to explore the impact of diverse biological environments on the surface characteristics of the samples, two representative DMEM and LB media were selected for this study. Figure 6e,f display the surface pH variations in Ti, PDA, MgCu-MOF74/Ti, and Ket@MgCu-MOF74/Ti samples after soaking in DMEM and LB medium for 6, 12, 24, 48, and 72 h. It can be observed that, after a period of time of immersing in both media due to the release of Mg ions, the MgCu-MOF74/Ti and Ket@MgCu-MOF74/Ti sample sets have different degrees of up-regulation in the first 24 h. Moreover, the surface pH of samples immersed in DMEM remained consistently higher than that of those in LB medium. Regardless of the medium, the results indicate the formation of an alkaline microenvironment on the surface of the MgCu-MOF74/Ti and Ket@MgCu-MOF74/Ti coatings.

The corrosion resistance of implant coatings is critical for their long-term success in biomedical environment. Corrosive degradation not only compromises the mechanical integrity and service life of orthopedic implants but also triggers adverse biological reactions by releasing metal ions and degradation products into the surrounding tissues [58,59]. Therefore, potentiodynamic polarization measurements were conducted to evaluate the protective efficacy of surface modification strategies [60,61]. The results confirm that PDA/MOF-based composite coatings can effectively suppress electrochemical activity and enhance corrosion resistance (See Supplementary Materials Figure S1 and Table S1).

### 3.3. Biological Properties of the Coating Samples

#### 3.3.1. Cell Biocompatibility

**Figure 7:**
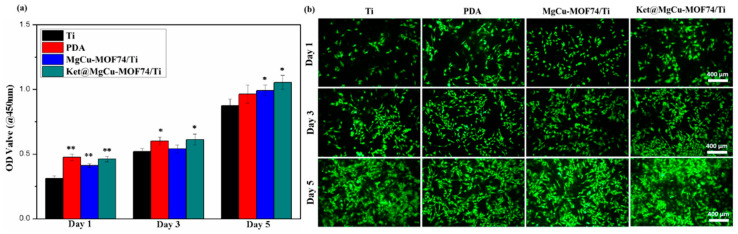
(**a**) Proliferation of SaOS-2 cells and (**b**) SaOS-2 cell proliferation measured by living cell staining, * *p* < 0.05 and ** *p* < 0.01 compared with Ti (*n* = 3).

The biocompatibility and cytotoxicity of implant materials are critical factors for their application in bone repair. In this study, the viability and proliferation of SaOS-2 osteoblast cells co-cultured with various material extracts were assessed qualitatively via live cell staining and quantitatively through the CCK-8 assay, respectively. The results, presented in Figure 7, demonstrate that cell proliferation followed this order: Ket@MgCu-MOF74/Ti ≈ MgCu-MOF74/Ti > PDA > Ti. The introduction of MgCu-MOF74 significantly enhanced the cellular activity and biocompatibility, underscoring its potential as a key factor for improving osteoblast proliferation. Notably, the performance of Ket@MgCu-MOF74/Ti was comparable to MgCu-MOF74/Ti, suggesting that the contribution of ketoprofen to cellular activity was relatively limited in this context. PDA-coated samples exhibited a significant stimulation of SaOS-2 cell proliferation within 1–5 days, which can be attributed to the excellent biocompatibility of the dopamine films. Furthermore, SaOS-2 viability exceeded 90% after five days of exposure, confirming that copper release does not elicit cytotoxic effects under our experimental conditions. In contrast, untreated Ti samples served as the control group and showed the lowest cell viability, highlighting the poor biocompatibility of pristine Ti materials. In conclusion, the results indicate that MgCu-MOF74 plays a pivotal role in enhancing cell viability and biocompatibility, while PDA coatings also contribute to improved cellular activity.

#### 3.3.2. Cell Differentiation

**Figure 8:**
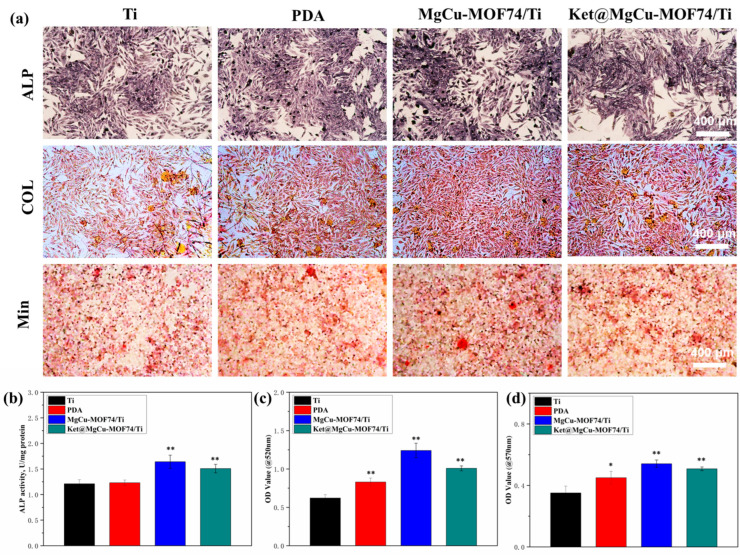
(**a**) The qualitative detection results of ALP activity, COL secretion, and mineralization of SaOS-2 cells, the quantitative results of (**b**) ALP activity, (**c**) COL secretion, and (**d**) mineralization in SaOS-2 cells, and * *p* < 0.05 and ** *p* < 0.01 compared with Ti (*n* = 3).

Figure 8 presents the results of ALP expression, collagen secretion, and mineralization levels for the different sample groups, revealing a progressive enhancement in osteogenic differentiation across Ti, PDA, Ket@MgCu-MOF74/Ti, and MgCu-MOF74/Ti. Both qualitative and quantitative analyses consistently demonstrated that the incorporation of the MOF coating plays a pivotal role in promoting osteogenic activity. This effect is primarily attributed to the controlled degradation of the MOF layer, which facilitates the sustained release of bioactive Mg^2+^ and Cu^2+^ ions, known for their osteogenic and angiogenic properties. Additionally, the presence of the PDA coating provided a favorable interface for cell adhesion and differentiation, further enhancing osteoblast function. Simultaneously, the results revealed that MOFs, when employed as drug carriers and chelated to the surface of titanium alloys via PDA, enable the precise and sustained release of the analgesic drug ketoprofen. This release profile was accomplished without compromising the coating’s osteogenic capacity, avoiding a reduction in new bone mineralization caused by drug overdose [40]. Overall, the findings underscore the importance of MgCu-MOF74 in stimulating osteogenic differentiation, providing a promising strategy for improving bone regeneration in implant materials.

**Figure 9:**
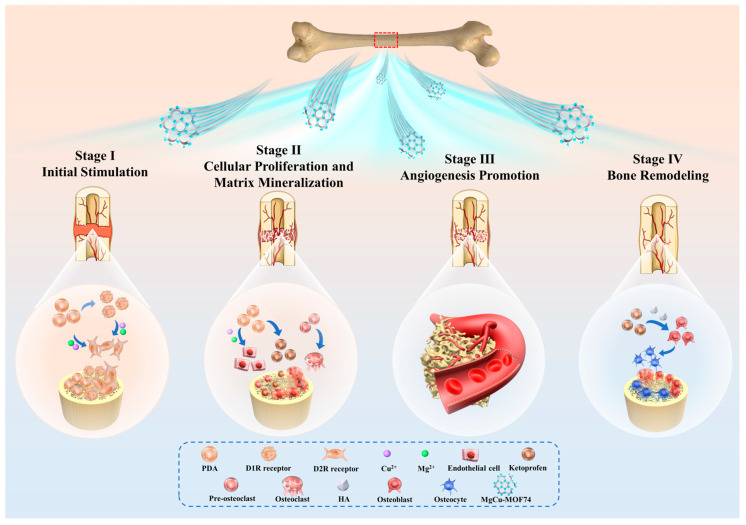
Schematic diagram of Ket@MgCu-MOF74/Ti promoting bone regeneration.

The mechanism of MgCu-MOF74 contributing to bone can be categorized into the following four stages [62,63,64], as shown in Figure 9. The bone regeneration process facilitated by MgCu-MOF74 can be summarized in four stages. Initially, the release of Mg^2+^ and Cu^2+^ ions creates a favorable microenvironment for osteogenesis, stimulating osteoblast activity, while PDA enhances bone formation by modulating osteoblast and osteoclast receptors [65,66]. In the second stage, these ions promote cellular proliferation, extracellular matrix synthesis, and collagen deposition, supporting bone matrix formation [67]. In the third stage, Cu^2+^ ions drive angiogenesis, improving blood supply, while Mg^2+^ ions enhance matrix mineralization, ensuring nutrient delivery for sustained bone growth [68]. Finally, in the remodeling phase, the balanced release of ions regulates osteoblast and osteoclast activity, maintaining structural integrity and accelerating bone repair with improved strength and quality [69,70]. Additionally, ketoprofen works by non-selectively inhibiting cyclooxygenase enzymes (COX-1 and COX-2), which are enzymes involved in the production of prostaglandins [71]. Previous in vivo animal studies in goats and rabbits demonstrated that the daily subcutaneous injection of ketoprofen at 2 mg/kg for several weeks did not affect mesenchymal stem cell proliferation or osteogenesis, indicating that ketoprofen does not inhibit bone healing [72]. Furthermore, the localized pH changes induced by MOF degradation could create a favorable environment for osteogenesis [73]. As presented in Figure 6, all samples maintained weakly alkaline microenvironments (pH 7.3–7.8), with the pH values following this sequence: Ti < PDA < Ket@MgCu-MOF74/Ti < MgCu-MOF74/Ti. This pH gradient was consistent with the observed osteogenic potential of each coating, indicating that the pH environment created by the degradation process correlates with bone-forming capacity.

### 3.4. Antibacterial Capacity

**Figure 10:**
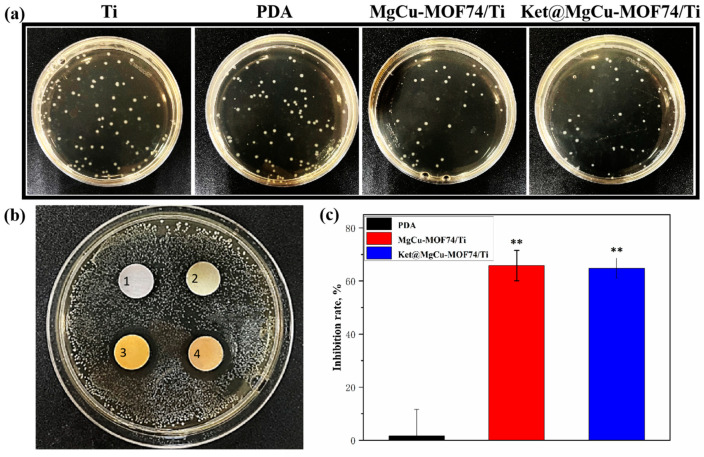
The (**a**) qualitative and (**c**) quantitative results of the coated plate test, (**b**) results of the inhibition circle, in which the inhibition zone markers 1, 2, 3, and 4 around different samples represent Ti, PDA, MgCu-MOF74/Ti, and Ket@MgCu-MOF74/Ti, ** *p* < 0.01 compared with Ti (*n* = 3).

The antibacterial properties of the different samples against *E. coli* are shown in Figure 10. Both qualitative results (Figure 10a) and inhibition zones (Figure 10b) demonstrate that MOF-modified samples (MgCu-MOF74/Ti and Ket@MgCu-MOF74/Ti) exhibit superior antibacterial activity compared to pure Ti and PDA-coated Ti, as further quantified in Figure 10c. The significant bacterial inhibition in MOF-modified samples can be attributed to the synergistic effects of bioactive ions (Cu^2+^, Mg^2+^, and OH^−^) released from the MOF structure. These ions are locally delivered at the bacteria interface, effectively disrupting bacterial cellular functions [74].

Cu^2+^ ions play a key role in bacterial inhibition by targeting thiol groups, destabilizing cell membranes, and inducing oxidative stress, which generates reactive oxygen species (ROS), leading to protein oxidation, DNA degradation, and bacterial cell death. Furthermore, Cu^2+^ can bind bacterial enzymes critical for cellular respiration and metabolism, impairing their function and viability [75,76]. The release of OH^−^ ions enhances this effect by altering the pH environment, leading to cell membrane damage and bacterial lysis. Although the pH values generated by Mg^2+^ release in the bacterial medium are not sufficient to directly inhibit *E. coli* proliferation according to established microbiological criteria [77,78], Mg^2+^ plays an important auxiliary role. It increases bacterial membrane permeability and disrupts cell wall stability, thereby potentiating the bactericidal actions of Cu^2+^ ions. These findings support that the overall antibacterial effect is primarily due to Cu^2+^, with Mg^2+^ serving as a synergistic cofactor [79]. Meanwhile, the Ket@MgCu-MOF74/Ti sample did not exhibit statistically significant improvement over MgCu-MOF74/Ti in antibacterial performance (Figure 10c). Notably, the release of Cu^2+^ and Mg^2+^ not only boosts antibacterial activity but may also impact osteogenic differentiation. In conclusion, the antibacterial efficacy of MOF-modified samples is primarily driven by the controlled release of antibacterial ions. Although Ket@MgCu-MOF74/Ti did not demonstrate a significant enhancement in antibacterial capacity, its anti-inflammatory properties may contribute synergistically to bacterial inhibition and tissue regeneration.

## 4. Conclusions

In this paper, MgCu-MOF74 powder was prepared by the hydrothermal method, followed by the loading of the anti-inflammatory drug ketoprofen into MgCu-MOF74. The results showed that the physicochemical properties of MgCu-MOF74 were stable. Subsequently, the Ket@MgCu-MOF74 powder was successfully bonded to the PDA layer on the titanium alloy surface by post-synthetic modification. The main conclusions are as follows:

Loading and release experiments showed that Ket@MgCu-MOF74 had a high loading capacity and well-controlled release ability for ketoprofen. After the treatment with PDA and MOF powder, the contact angle of the coating surface was significantly reduced. CCK-8 assays and live cell staining demonstrated that the new composite coating was non-cytotoxic. The results of the osteogenic experiment showed that MgCu-MOF74/Ti and Ket@MgCu-MOF74/Ti can significantly promote alkaline phosphatase activity, collagen secretion, and mineralization. MgCu-MOF74 coating loaded with ketoprofen enables the co-release of Mg^2+^ and Cu^2+^, generating an alkaline microenvironment at the implant surface that contributes to robust antibacterial efficacy. More importantly, the incorporation of Cu^2+^ not only enhances the antibacterial functionality through membrane disruption and oxidative stress induction but also significantly improves the aqueous stability and controlled ion release behavior of the MOF under physiological conditions. These improvements effectively address the known limitations of conventional Mg-MOF74, such as poor hydrolytic stability and limited bioactivity, and confirm the synergistic role of Cu^2+^ in promoting both osteointegration and anti-infective performance, thereby demonstrating a substantial advancement over prior Mg-MOF-based systems.

## Data Availability

The data presented in this study are available on request from the corresponding author.

## Supplementary Materials

The following supporting information can be downloaded at https://www.mdpi.com/article/10.3390/jfb16060222/s1. Figure S1: Polarization curves of various specimens; Table S1: Electrochemical parameters for various specimens [41,58,59,60,80].
