# Supplementary material for: Enhanced Osteogenesis and Antibacterial Properties of Ketoprofen-Loaded MgCu-MOF74-Coated Titanium Alloy for Bone Implant
Source: J Funct Biomater. 2025 Jun 14;16(6):222. doi: 10.3390/jfb16060222 (PMC12194423; doi:10.3390/jfb16060222)
Supplement: Supplementary file 1 [file jfb-16-00222-s001.zip › jfb-3644265-supplementary.pdf]

The electrochemical stability and corrosion resistance of titanium-based implant coatings are of paramount importance for their successful long-term performance in biomedical environments. Corrosive degradation not only compromises the mechanical integrity and lifespan of orthopedic implants, but also induces adverse biological responses by releasing metal ions and degradation products into the surrounding tissue [1,2]. Therefore, comprehensive electrochemical testing, including potentiodynamic polarization and quantitative measurement of corrosion parameters, is essential to assess the protective efficacy of surface modification strategies, such as MOF-based coatings, under physiologically relevant conditions [3,4].

The electrochemical experiments were supplemented to evaluate the corrosion resistance and interfacial electrochemical behavior of different coatings. The experiment was performed using an electrochemical workstation (CS31OH, Potentionstat/Galvanostat, China) equipped with a three - electrode electrochemical cell. In this study, the workstation was configured with a platinum counter electrode, a saturated calomel electrode (SCE) as the reference electrode, and the coated sample as the working electrode. All electrodes were immersed in the corrosive solution. Before the aforementioned tests, the test samples were first maintained under open - circuit potential (OCP) for 30 minutes to ensure potential stability. Specifically, potentiodynamic polarization tests were performed on Ti, PDA, MgCu-MOF74/Ti, and Ket@MgCu-MOF74/Ti samples in the 0.9% NaCl solution.

The electrochemical parameters, including corrosion potential (E_{corr}) and corrosion current density (i_{corr}), are tabulated in Table S1. As shown in Table S1 and Figure S1, the MgCu-MOF74/Ti and Ket@MgCu-MOF74/Ti coatings both exhibit improved corrosion resistance compared to bare Ti and PDA-modified Ti. The E_{corr} value of MgCu-MOF74/Ti shifts positively to -0.167 ± 0.007 V, indicating a more noble corrosion potential, while the i_{corr} value is markedly reduced to $(3.86 \pm 1.10) \times 10^{-8}$ A/cm², nearly two orders of magnitude lower than that of pure Ti ($(2.01 \pm 0.124) \times 10^{-6}$ A/cm²). Similarly, Ket@MgCu-MOF74/Ti demonstrates an extremely low i_{corr} of $(3.72 \pm 0.657) \times 10^{-8}$ A/cm². These results confirm that the MOF-based composite coatings effectively suppress the electrochemical activity and enhance the passivation of the titanium substrate. This is primarily attributed to the conformal and stable MOF coating formed by dopamine-assisted deposition, which not only provides a physical barrier but also regulates ion release to maintain the stability of the interface under physiological conditions [5].

Our findings are consistent with previous studies, which have shown that the introduction of bioactive MOF coatings on titanium alloys can improve corrosion resistance and long-term electrochemical stability in biomedical environments [4]. The enhanced corrosion protection provided by composite coatings is essential for maintaining the structural integrity and biological function of orthopedic implants *in vivo*.

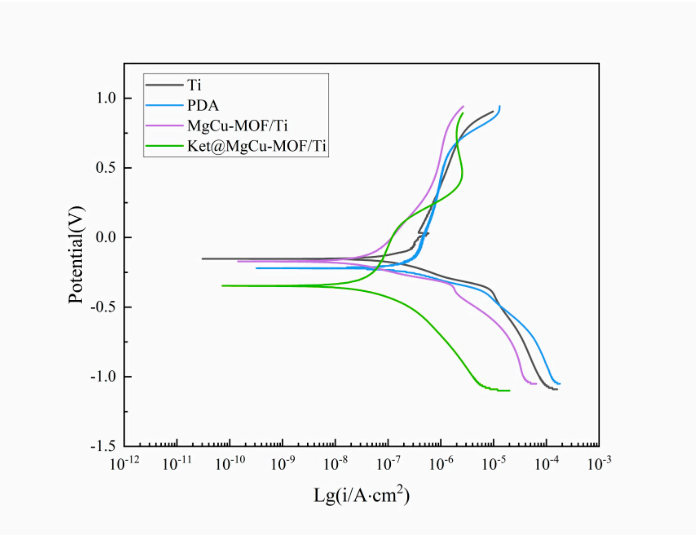

Figure S1 Polarization curves of various specimens

Table S1 Electrochemical parameters for various specimens

Samples	Ti	PDA	MgCu-MOF/Ti	Ket@MgCu MOF/Ti
E_{corr} (V)	−0.273 ± 0.021	−0.240 ± 0.019	−0.167 ± 0.007	−0.3187 ± 0.043
i_{corr} (A/cm^2)	(2.01 ± 0.124) × 10^{-6}	(3.69 ± 0.462) × 10^{-7}	(3.86 ± 1.10) × 10^{-8}	(3.72 ± 0.657) × 10^{-8}

References

1. Geetha, M.; Singh, A.K.; Asokamani, R.; Gogia, A.K. Ti based biomaterials, the ultimate choice for orthopaedic implants—A review. *Prog. Mater. Sci.* **2009**, *54*, 397–425.
2. Wang, J.; Zhang, S.; Sun, Z.; Wang, H.; Ren, L.; Yang, K. Optimization of mechanical property, antibacterial property and corrosion resistance of Ti-Cu alloy for dental implant. *J. Mater. Sci. Technol.* **2019**, *35*, 2336–2344.
3. Li, A.; Wang, Q.; Chen, R.; Ding, X.; Su, Y.; Fu, H. Application of alloying for enhancing the corrosion resistance of titanium alloys: A review. *Mater. Today Commun.* **2025**, *42*, 111111.
4. Zhang, Y.; Cheng, Z.; Liu, Z.; Shen, X.; Cai, C.; Li, M.; Luo, Z. Functionally tailored metal—organic framework coatings for mediating Ti implant osseointegration. *Adv. Sci.* **2023**, *10*, 2303958.
5. Ho, C.-C.; Ding, S.-J. Novel SiO_2/PDA hybrid coatings to promote osteoblast-like cell expression on titanium implants. *Journal of Materials Chemistry B* **2015**, *3*, 2698–2707.
